# Content validity of a novel patient-reported and observer-reported outcomes assessment to evaluate ocular symptoms associated with infectious conjunctivitis in both adult and pediatric populations

**DOI:** 10.1186/s12955-019-1223-9

**Published:** 2019-10-30

**Authors:** Sujata P. Sarda, Marie De La Cruz, Emuella M. Flood, Magdalena Vanya, David G. Hwang, Christopher N. Ta, Abhijit Narvekar

**Affiliations:** 1 Shire, a Takeda company, 300 Shire Way, Lexington, MA 02421 USA; 2 ICON PLC, Gaithersburg, MD 20878 USA; 3 ICON PLC, 601 Gateway Blvd Suite 1250, South San Francisco, CA 94080 USA; 4 Stanford University School of Medicine, Byers Eye Institute at Stanford, 2452 Watson Court, Palo Alto, CA 94303 USA; 5 Cornea Service, Department of Ophthalmology, University of California, San Francisco School of Medicine, San Francisco, CA 94143-0730 USA

**Keywords:** Bacterial conjunctivitis, Observer-reported outcome measure, Patient-reported outcome measure, Viral conjunctivitis

## Abstract

**Background:**

Acute infectious conjunctivitis is a common condition most frequently caused by viruses or bacteria. Clinical outcome assessments have been used to assess signs and symptoms of bacterial and viral conjunctivitis, but have not been evaluated for content validity. We aimed to develop content-valid patient- (PRO) and observer-reported outcome (ObsRO) instruments to assess symptoms of ocular discomfort associated with viral or bacterial conjunctivitis in adult and pediatric patients.

**Methods:**

Draft items were developed from a previous review of published studies from 2001 to 2015. Patients and caregivers of patients with a diagnosis of viral or bacterial conjunctivitis within the past 6 months were recruited. Concept elicitation with open-ended questions explored signs and symptoms, followed by cognitive interviewing to assess clarity and relevance of the draft items. Patients aged ≥8 years were interviewed for the PRO; parents/caregivers of children aged 1–10 years were interviewed for the ObsRO. Interviews were conducted in three rounds to allow changes. Concept saturation was documented using a saturation grid. Cognitive interview data were analyzed iteratively and focused on clarity, relevance and inconsistent interpretation of the instrument’s content.

**Results:**

Overall, 23 patients or parents/caregivers participated (round 1, *n* = 10; round 2, *n* = 6; round 3, *n* = 7). Data saturation was reached by the 16th interview. The most frequent spontaneously reported signs/symptoms were: discharge, red/pink eyes, itchiness, swelling/puffiness, watery eyes, pain, burning and foreign body sensation. Itching, pain/burning/stinging and foreign body sensation were most commonly reported as the top three most bothersome symptoms. Interview results indicated that items on pain, itching and foreign body sensation for the PRO and pain or discomfort for the ObsRO were relevant to the patients’ experience of conjunctivitis and were clear and easy to understand.

**Conclusions:**

PRO and ObsRO items were found to be clear, relevant and appropriate in assessing key viral and bacterial conjunctivitis symptoms in adult and pediatric patients.

## Background

Acute infectious conjunctivitis is a commonly encountered disorder in children and adults in both primary care and specialty eye care settings. The predominant causes of infectious conjunctivitis are viral and bacterial pathogens; other causes are much rarer [1]. Infectious conjunctivitis typically presents as a red eye with discharge [1] and is usually self-limiting, but in rare cases can lead to complications such as keratitis and blindness [2, 3]. Infectious conjunctivitis may impose both economic and social burden upon affected individuals, caregivers and contacts in the workplace, school or household [1]. The economic impact can include the direct costs of medical evaluation, treatment, supportive care and isolation precautions, as well as the indirect costs of missed work or school days and lost productivity of affected individuals and caregivers [4]. The estimated direct cost of conjunctivitis is approximately $800 million per year in the United States [4].

Clinical outcome assessments (COAs) are used to measure symptoms, overall mental state and patient functioning in drug development programs to capture treatment benefit and support regulatory approval [5]. COAs used in this context must be well defined and reliable and have evidence of content validity, as described in guidance from the US Food and Drug Administration (FDA) on the development and use of patient-reported outcomes (PROs) [5]. COAs include PROs, observer-reported outcomes (ObsROs), clinician-reported outcomes (ClinROs) and performance outcomes (PerfOs) [6]. PROs are used to assess symptoms or other concepts only known and best reported by the patient, whereas ObsROs are used in cases where patients are unable to self-report signs and symptoms, such as in young children, and for which no special training is required for the assessment [6]. PROs [2, 7–9], ObsROs [2, 9] and ClinROs [2, 7–12] have been used in published studies to assess clinical signs and symptoms of bacterial and viral conjunctivitis. However, based on a review of published articles from 2001 to 2015, there have been no reports of PROs or ObsROs used for assessing bacterial and viral conjunctivitis that were developed according to the FDA’s PRO guidance, i.e., the content validity of the instruments were not evaluated in the target population through cognitive interviewing (ICON PLC, data on file). In addition, some of the articles reported that ClinROs had been used to assess symptoms as well as signs, even though symptoms are best reported by patients directly [6].

Given that published COAs for acute infectious conjunctivitis captured in our literature search lacked documentation of their development according to FDA guidelines for reliability and content validity, we intended to develop a content-valid PRO and ObsRO for use in clinical trials of adenoviral and bacterial conjunctivitis to assess changes in symptoms of ocular discomfort. In this study, two instruments were developed to assess ocular discomfort symptoms associated with acute infectious conjunctivitis: a PRO for adults and children aged ≥8 years and an ObsRO for children aged < 8 years. This paper describes the qualitative evaluation of the novel PRO and ObsRO to support their content validity and potential application to clinical research conducted in children and adults with acute infectious conjunctivitis.

## Methods

**Fig. 1:**
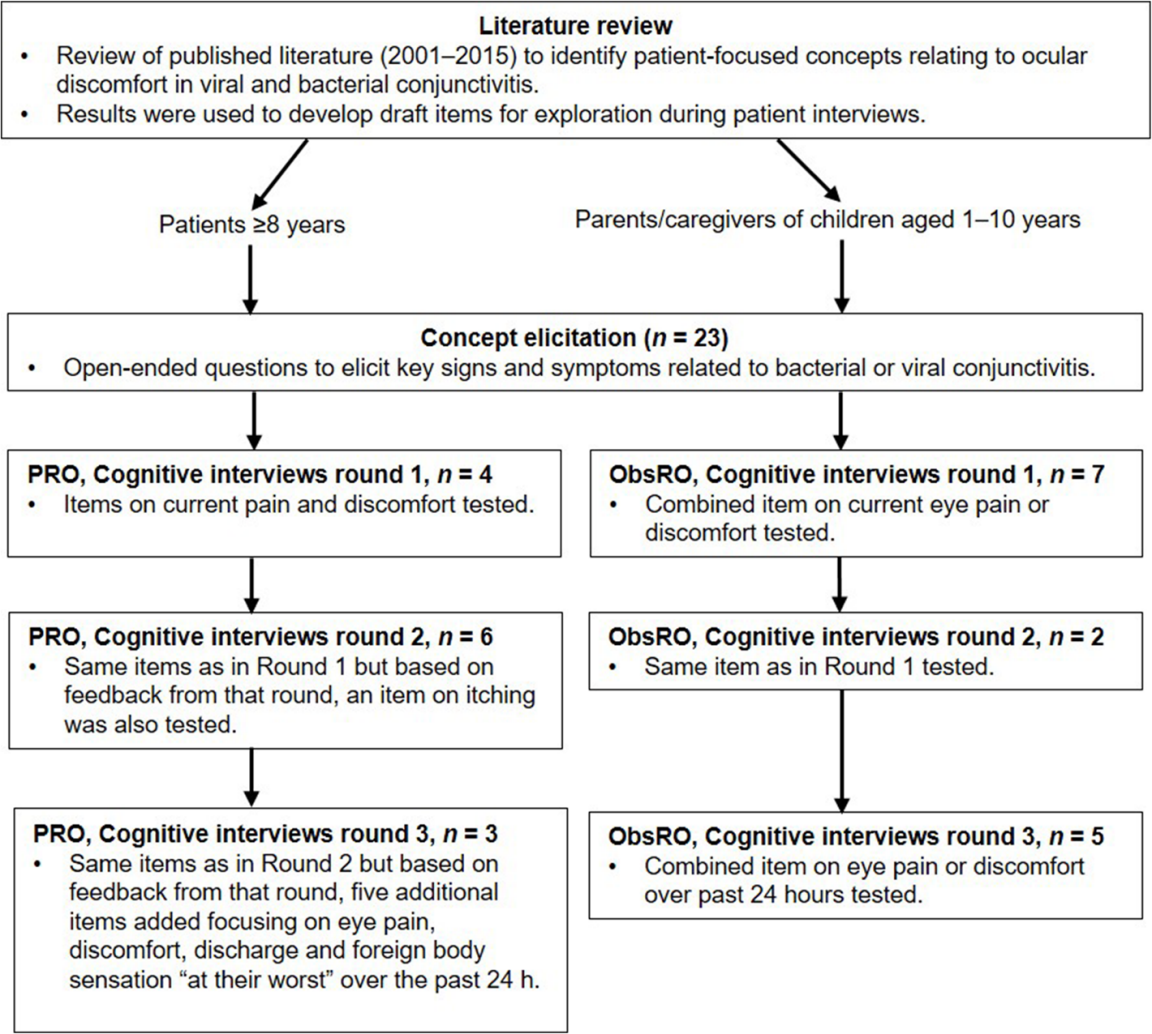
Study flow. The interviews combined both concept elicitation and cognitive interviewing. After each round of cognitive interviewing, the instruments were refined and tested in a new set of patients. *ObsRO* Observer-reported outcome; *PRO* Patient-reported outcome

The study flow for the development of the PRO and ObsRO instruments are summarized in Fig. 1.

### Initial drafting of the PRO and ObsRO items

Draft items were developed based on a previous review of the published literature for articles from 2001 to 2015 to identify relevant patient-focused concepts (especially relating to patient-reported discomfort) for assessing treatment benefit in patients with viral and bacterial conjunctivitis. Results from the literature review showed that ocular discomfort and pain were common patient-reported concepts in bacterial and viral conjunctivitis (ICON PLC, data on file).

The final instrument was intended to assess ocular pain and discomfort during periodic study visits in clinical trials of adenoviral and bacterial conjunctivitis. The initial draft instrument utilized an 11-point numeric rating scale, a commonly used scale to capture pain, and was developed as a current assessment rather than incorporating a recall period. An adaptation of the instrument was developed for use by parents and/or other caregivers (henceforth referred to as “caregivers”) in patients aged < 8 years (ObsRO). For the PRO, multiple draft items were developed for exploration during patient interviews to evaluate different options for capturing symptoms, such as whether pain and discomfort should be combined into one item or separated into different items. For the ObsRO, one item was drafted to capture eye pain or discomfort based on observable behaviors. These candidate items were tested during the combined concept elicitation and cognitive interviews.

### Study design

Written informed consent and assent were obtained from adult patients/caregivers and child/adolescent patients, respectively. The study involved one-time, one-on-one, face-to-face interviews with patients and/or caregivers of patients with bacterial or viral conjunctivitis. The interviews combined both concept elicitation and cognitive interviewing. In the concept elicitation portion, participants were asked to recall their experience of conjunctivitis (focusing on ocular discomfort) that occurred within the prior 6 months in order to identify key concepts (i.e., signs, symptoms and impacts) important to patients with conjunctivitis. The cognitive interviewing portion was used to assess the clarity and relevance of the draft items. All interviews lasted ~ 60 min and were conducted in three rounds, each of which had unique participants. After each round, changes were made to the PRO and ObsRO (caregiver-reported) items, and the revised instrument was tested in the subsequent round. There were no prespecified rules guiding revision of the instruments after each round; decisions were based on the data findings and the judgment of the study team. Three drafts of the questionnaires (one per round) were written before the PRO and ObsRO instruments were finalized. The PRO and ObsRO were developed concurrently but independently of each other.

#### Recruitment of participants

Patients and/or caregivers of patients with a diagnosis of viral or bacterial conjunctivitis within the prior 6 months were recruited from the Baltimore, MD metropolitan area of the United States. Patients were required to be free of symptoms of conjunctivitis for ≥1 week before the interview and to have oral and written fluency in English. Patients aged ≥8 years completed the PRO; caregivers of children aged 1–10 years completed the ObsRO. A total of 25 patients were targeted, with the aim of including ≥5 patients within each of the following subgroups: (1) caregivers of infants/toddlers aged 1–3 years; (2) caregivers of children aged 4–7 years; (3) dyads of children aged 8–10 years and their caregivers; (4) children/adolescents aged 11–17 years; and (5) adults aged ≥18 years.

Individuals with any serious psychiatric disorder or other condition that could interfere with providing informed consent/assent or participating in an interview to discuss health were excluded from the study. Potential patients were identified using a patient database provided by the firm Global Perspectives (Norwich, UK), through social media and/or via direct referral solicitation. Potentially eligible patients or caregivers of patients were screened by Global Perspectives. Diagnosis was based on a physician-completed diagnosis form (*n* = 2), discharge documents or visit summaries (*n* = 6), copies of the paper prescription (*n* = 13), physician notes (*n* = 1) or diagnosis information from a participant’s health insurance or pharmacy online portal (*n* = 1). Best efforts were made to recruit a diverse sample based on sex and race/ethnicity.

#### Concept elicitation

Participants were asked open-ended questions about their conjunctivitis signs and symptoms and experiences related to bacterial or viral conjunctivitis within the past 6 months. After allowing the patient to describe his or her symptoms spontaneously, the interviewer then probed for specific terms relating to conjunctivitis signs and symptoms. These included discomfort, pain, pressure, itching, burning, feeling like something is in the eye (foreign body sensation), watery eyes and discharge. Interviewers then asked each patient to rank the top three most bothersome signs or symptoms. Caregivers were asked to rank the top three most bothersome signs and symptoms based on what they had observed and/or heard from their child. This was followed by questions on the impact of conjunctivitis on the patient. If a symptom/sign was reported by both parent and child in a dyad, it was counted once. If only one individual in the dyad reported a sign/symptom, that was also counted once.

#### Cognitive interviewing

In round 1 of the PRO, items on current pain and discomfort were tested. These included a question asking about eye discomfort or eye pain, 2 questions querying eye pain and discomfort separately and separate questions on eye discomfort and eye pain with additional descriptions for pain (hurting, burning or stinging feeling) and discomfort (feeling of pressure, itchiness, foreign body sensation or the eye feeling uncomfortable). In round 2, patients completed the same questions as in round 1 but with the addition of one question based on feedback from the round 1 interviews. In round 3, five additional questions were added based on feedback from round 2 and a decision by the investigators to test the items with a 24-h recall period. The 5 additional items focused on the symptom “at its worst” over the past 24 h.

Caregivers in round 1 of the ObsRO were asked to complete one item asking if their child was currently showing observable signs of pain or discomfort, such as eye rubbing, tearing or child expressing pain or discomfort. Only one item was included for the ObsRO because these signs would likely be relevant to all symptoms associated with pain or discomfort. In round 2, caregivers were asked to complete the same item used in round 1. In round 3, the ObsRO was revised to include a 24-h recall.

#### Qualitative analysis

All interviews were audio recorded and transcribed verbatim; transcripts were reviewed to remove identifying data and correct any transcription errors. All transcripts were coded using MaxQDA v.11 (VERBI Software GmbH, Berlin, Germany). For the concept elicitation portion of the interviews, the data were assessed to document concept saturation, the point at which no new concepts arose during the course of the interviews. A saturation grid was developed to document concept saturation. Signs and symptoms mentioned by patients or caregivers when responding to open-ended questions were coded as spontaneous reports. For the cognitive interviewing portion, the draft PRO and ObsRO items were evaluated for the clarity of their instructions, applicability of their response options and understanding of item content. Cognitive interview data were analyzed iteratively, with data organized by question to allow for identification of issues of clarity, relevance or inconsistency in interpretation. Descriptive statistics (e.g., mean, range, frequency) were used to describe patients’ sociodemographic information.

## Results

### Participants

Overall, 23 patients or caregivers were recruited across three rounds of interviews (Table 1). Almost all interviews were conducted in June 2016, with one interview on 1 July 2016. Key demographics of the participants are shown in Table 2. All adult patients were female (*n* = 5), and the majority (*n* = 3/5) were black. One-half (*n* = 2/4) of all child/adolescent patients were black and one-half were female. The majority of caregivers were female (*n* = 9/10) and black (*n* = 6/10), and all cared for a child aged 1–6 years. The caregivers in the dyads were all female and most (*n* = 3/4) were white, while the children in the dyads were aged 9–10 years and had completed the fourth grade. The duration of conjunctivitis experienced by patients ranged from 2 days to a week and a half. The majority (*n* = 14) of patients had bilateral conjunctivitis, while 9 developed conjunctivitis in only one eye.

**Table 1:** Number of patients in each round

Participant	Round 1 *n* = 10	Round 2 *n* = 6	Round 3 *n* = 7
Adult	1	3	1
Child/adolescent	2	1	1
Caregiver	6	–	4
Caregiver/child dyad	1	2	1

**Table 2:** Demographics of the participants

Characteristic	Adult patients *n* = 5	Child/adolescent patients *n* = 4	Caregivers of children *n* = 10	Caregiver/child dyads *n* = 4
Age, years, mean (range)	56 (44–70)	14 (12–15)	38 (28–62)	Caregivers: 40 (32–44) Children: 10 (9–10)
Female, *n* (%)	5 (100)	2 (50)	9 (90)	Caregivers: 4 (100) Children: 1 (25)
Race, *n* (%)				
Black	3 (60)	2 (50)	6 (60)	Caregivers: 1 (25) Children: 1 (25)
White	2 (40)	2 (50)	3 (30)	Caregivers: 3 (75) Children: 3 (75)
Asian	0	0	1 (10)	0
Highest level of education, *n* (%)				
High school	1 (20)	–	0	Caregivers: 0
Some college	0	–	3 (30)	Caregivers: 1 (25)
Associate degree	2 (40)		1 (10)	Caregivers: 1 (25)
Bachelor’s degree	2 (40)	–	4 (40)	Caregivers: 0
Master’s/doctoral	0	–	2 (20)	Caregivers: 2 (50)

### Concept elicitation findings

**Fig. 2:**
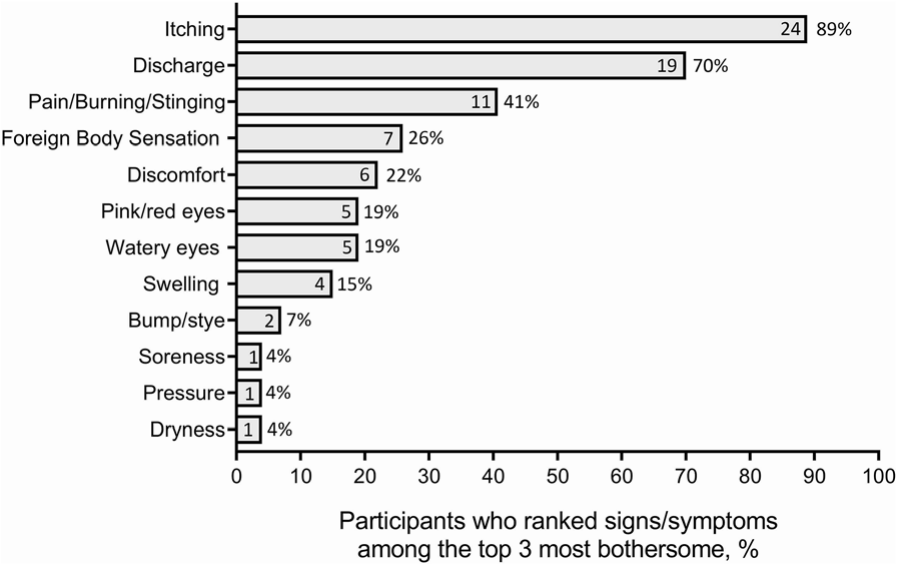
Frequency of signs/symptoms when respondents were asked to rank the three that are most bothersome. Values inside bars indicate number of patients with sign or symptom. Denominator = 27 (parents and children in dyads questioned separately)

Data saturation was reached by the 16th participant. Overall, patients and caregivers (*N* = 23) reported a total of 17 signs and symptoms, with an average of 7.8 signs/symptoms per patient. The most frequently reported signs and symptoms experienced by patients are shown in Table 3. These were either spontaneous responses to an open-ended question, or responses to a probe from the interviewer. The most frequent spontaneously reported signs/symptoms were discharge (*n* = 22/23), red/pink eyes (*n* = 21/23), itchiness (*n* = 15/23), swelling/puffiness (*n* = 12/23), watery eyes (*n* = 7/23), pain (*n* = 7/23), burning (*n* = 5/23), and foreign body sensation (*n* = 5/23). When participants were asked to rank the top three most bothersome signs or symptoms, the most frequently reported symptoms were itching, pain/burning/stinging and foreign body sensation; the most frequently reported signs were discharge, pink/red eyes and bump/stye (Fig. 2). Most adult (*n* = 4/5) and child/adolescent (*n* = 7/8) patients reported impacts of conjunctivitis on daily activities, including missing work/school and social interactions with family and friends. Emotional impacts, such as feeling annoyed, irritated, bored and lonely, were also reported by patients. All 14 caregivers reported impacts of conjunctivitis on their child with respect to daily activities and/or emotional impacts.

**Table 3:** Most frequently reported concepts in concept elicitation interviews (*N* = 23)

Concept	Participants reporting concept, *n*	Spontaneous, % participants reporting concept^a^	Probed, % participants reporting concept^a^	Other terms used to describe concept	Example quote (participant)
Discharge	22	100	0	Crust/crusty/crusting (*n* = 6), yellow (*n* = 4), like something was in the eye (*n* = 3), gooey (*n* = 3)	… I got up in the morning, my eye was like, like crusted shut … so I’d have to really get the cotton balls, put them on, you know, wet them and kind of wipe the stuff out of my eye, and that was like probably every 15, 20 min (adult patient)
Red/pink eyes	22	95	5	Pink (*n* = 5), red (*n* = 4), irritated (*n* = 4)	Actually, she started rubbing her eyes. And then I noticed her eye was redder than the other eye (caregiver)
Itching/Itchiness	22	68	32	Rubbing eyes (*n* = 11), itch/itches/itching (*n* = 10), irritated/irritation/irritating (*n* = 4)	… It would itch a little bit so I would rub my eye and then it would just keep getting worse and worse...I wanted to like do something but there was really nothing that I could do (child patient)
Swelling/puffiness	12	100	0	Puffy/puffiness (*n* = 7), eye bags (*n* = 2)	Yeah, puffy, swollen. A mess, wore sunglasses to go to weddings (adult patient)
Watery eyes	17	41	59	Cry/crying (*n* = 3), tears (*n* = 2), runny/running (*n* = 2), watery (*n* = 2), glassy/glossy (*n* = 2)	… kind of irritating … I had runny eyes and in between the mucus cleaning up, my eyes were running because I was constantly blinking … (adult patient)
Pain	13	54	46	Hurt/hurting (*n* = 5), burning (*n* = 4), itchy/itching (*n* = 3)	It felt like something was in your eye or like just – it was like stinging a little bit and it wasn’t that bad but it just hurt and you could tell that it was hurting (child patient)
Burning	12	42	58	Burned (*n* = 3), uncomfortable (*n* = 2), irritated/irritating (*n* = 2)	That’s like it wasn’t where it is like you slide some kind of chemical in your eye. It wasn’t like that type of burning, but it was like an irritating type of burn (adult patient)
Foreign body sensation	18	28	72	Like something is in eye (*n* = 9), itch/itching/itchiness (*n* = 6), scratchy (*n* = 2), constantly picking at the eye (*n* = 2), eyelash in/on the eye (*n* = 2)	… like if you get a piece of sand in your eye or something like that. Kind of scratchy I guess (adult patient)
Discomfort	21	10	90	Itchy/itching (*n* = 7), like something in the eye (*n* = 6), uncomfortable (*n* = 4)	Um, more of discomfort, um, because she wanted to rub her eye a lot. She just kept saying it feel like something’s in my eye (caregiver)

^a^Percentage of participants who reported each concept either spontaneously in response to an open-ended question or after probing by the interviewer

### Cognitive interview findings

**Fig. 3:**
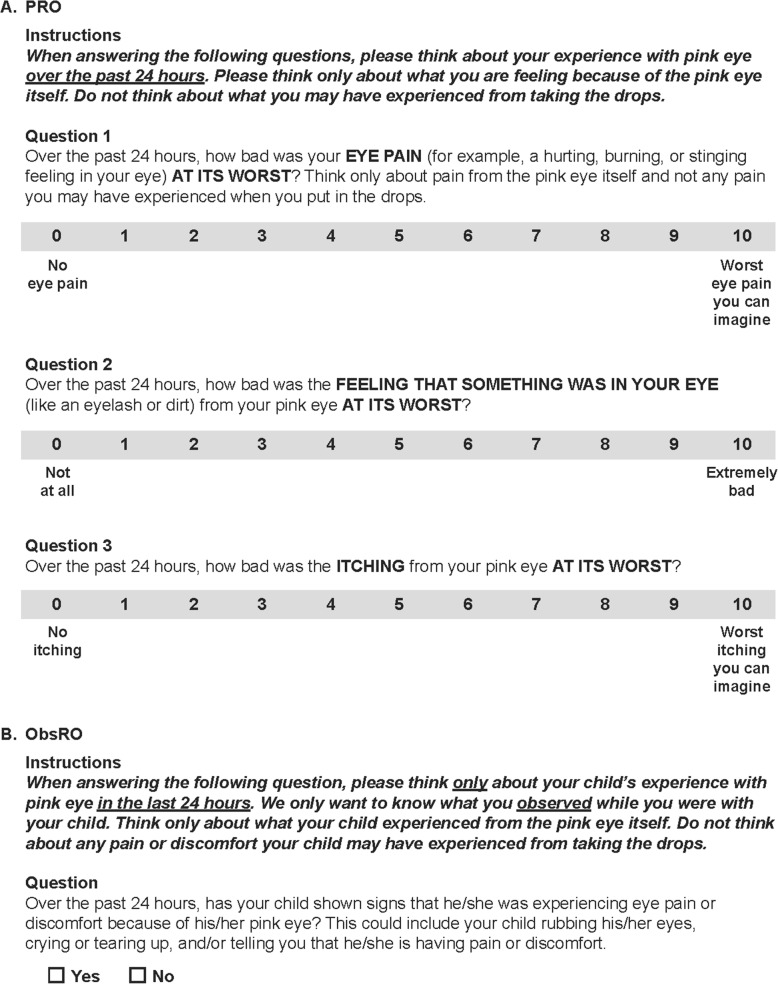
Full version of the conjunctivitis ocular discomfort scales (PRO and ObsRO). *ObsRO* Observer-reported outcome; *PRO* Patient-reported outcome

A summary of the issues and solutions during the cognitive interviews is shown in Table 4. The final versions of the PRO and ObsRO instruments are given in Fig. 3.

**Table 4:** Summary of issues and solutions during the cognitive interviews

Issue	Category	Solutions
Round 1 PRO		
Interpretations of “eye discomfort” were highly variable across patients	Inconsistency in interpretation	Item retained without modification for further testing in round 2
There was disagreement on whether pain and discomfort represented the same concept or should be assessed separately	Inconsistency in interpretation	Item retained without modification for further testing in round 2
Itching commonly reported in concept elicitation	Relevance	Item on itching added for testing in round 2
Round 2 PRO		
Foreign body sensation and discharge were commonly reported in concept elicitation	Relevance	Items on foreign body sensation and discharge added for testing in round 3
Participants provided various recall periods when answering questions. There was also variability in symptom severity over the course of the day	Clarity in recall period	24-h recall period added for testing in round 3; participants were asked to recall symptoms/signs at their worst over a 24-h period
Round 3 PRO		
Discharge commonly reported in concept elicitation	Relevance	Item on discharge added for testing in round 3, but on completion of this round, discharge was removed from the final PRO to focus on symptoms only
Interpretations of “eye discomfort” were highly variable across patients	Inconsistency in interpretation	Item removed from the final PRO
Round 1 ObsRO		
None	–	–
Round 2 ObsRO		
As there was variability in symptom severity over the course of the day, a 24-h recall was recommended	Clarity in recall period	24-h recall period added for testing in round 3; participants were asked to recall symptoms/signs over 24-h period
Round 3 ObsRO		
None	–	–

*ObsRO* Observer-reported outcome, *PRO* Patient-reported outcome

### PROs

Overall, the response options (0–10 scale, 0 = not at all, 10 = extremely bad/worst) in the three PRO rounds were found to be clear, easy to understand and appropriate. Results from the round 1 interviews suggested that eye pain and discomfort were relevant to the experience of conjunctivitis in these patients. When they were asked what they thought about the examples of eye pain provided (i.e., hurting, burning or stinging feeling in the eye), the patients generally found the examples to be helpful and appropriate and this was also the result in rounds 2 and 3. However, definitions of eye discomfort were highly variable across patients, and there was disagreement as to whether pain and discomfort represented the same concept or should be assessed separately. Round 1 interviews also showed that itching was an important and common symptom for conjunctivitis patients. Therefore, an item on itching was added to the PRO and tested in round 2. When the patients were asked what they thought about the examples of eye discomfort provided (i.e., feeling of pressure, itchiness or like something is in your eye or your eye feeling uncomfortable), the patients generally found the descriptions to be helpful and appropriate, with the same result in rounds 2 and 3.

Round 2 interviews confirmed the importance of itching. Data in round 2 also suggested that foreign body sensation and discharge were relevant and important concepts; therefore additional items on these two concepts were added in round 3 for further exploration. When patients were asked to explain the meaning of the question “how bad is your eye pain or discomfort from your pink eye?” there were mixed interpretations. Three of the 6 patients defined the questions as asking about both discomfort and pain, 1 patient defined the question as how the eye felt overall, 1 patient discussed discomfort only and another discussed pain only.

After round 2, it was decided not to remove any items so these could be tested further in round 3. In addition, five items were added on pain, discomfort, itching, discharge and foreign body discharge “at their worst” in the “past 24 h.” The 24-h recall and “symptoms/signs at their worst” wording were added to account for any variability in symptom severity over the course of the day. Discharge was introduced in round 3 because it was a common concept often mentioned during the concept elicitation interviews in rounds 1 and 2. Round 3 results provided further support for the key concepts of pain, itching and foreign body sensation. Items in round 3 were considered clear and easy to answer using the 24-h recall. After round 3, it was decided to focus on symptoms only as clinicians would evaluate signs. As a result, discharge was removed. To improve clarity, the pain item was retained and defined as hurting, burning or stinging. The discomfort item was removed due to inconsistent interpretation by patients.

### ObsROs

In round 1 and 2, caregivers were asked if their child was showing observable signs of pain or discomfort, such as eye rubbing, tearing or child expressing pain or discomfort. In round 1, the majority (*n* = 6/7) of caregivers reported the question to be easy to understand, and all caregivers correctly interpreted the question and found it relevant to their child’s experience with conjunctivitis. In round 2, the 2 caregivers reported the question as easy to understand and correctly interpreted its meaning, and found the question relevant. In round 3, all 5 caregivers understood the meaning of the question and found it relevant when asked with a 24-h recall. As with the PRO, the 24-h recall period had been added at round 3 to account for variability over the course of the day.

## Discussion

In this study, we developed novel PRO and ObsRO instruments to assess symptoms of ocular discomfort associated with viral or bacterial conjunctivitis in adult and pediatric patients. The focus of the final instruments is on symptoms because clinicians would be tasked with evaluating signs such as discharge and redness. Our results suggest that pain, itching and foreign body sensation are key symptoms of viral and bacterial conjunctivitis and support their inclusion in the PRO using a 0–10 scale, while a single item with a binary (yes/no) response on observable signs of pain or discomfort is suitable for the ObsRO. The term “discomfort” was removed from the PRO due to inconsistent interpretation by patients, but “ocular discomfort” can still be used to collectively describe these symptoms. Overall, the PRO and ObsRO items were found to be clear, relevant and appropriate in assessing key symptoms of viral and bacterial conjunctivitis in adult and pediatric patients.

To our knowledge, this is the first study to develop an ObsRO and to assess content validity of a PRO and an ObsRO for acute infectious conjunctivitis in both adult and pediatric patients. The results of a systematic literature search suggest that the symptoms of viral and bacterial conjunctivitis are generally similar [13], so the PRO and ObsRO should apply to both bacterial and viral conjunctivitis. Another finding to note from this study was that conjunctivitis was associated with a wide range of impacts on patients’ everyday functioning, particularly social interactions with family and friends for both adults and children.

The instruments developed in this study are intended to be used to evaluate ocular discomfort symptoms in patients with adenoviral and bacterial conjunctivitis. For the PRO, patients are asked to think only about what they are feeling because of the conjunctivitis itself and not about what they have experienced from taking the drops. This also applies to caregivers administered the ObsRO when thinking about their child’s experience with conjunctivitis. The PRO and ObsRO take < 5 min to administer and respondent burden is minimal, making it easier to implement in trials or real-world clinical practice. In addition, for patients that are not able to read or write, versions of the PRO and ObsRO that can be administered entirely by interviewers have also been developed. Analyses from clinical trial data will help to examine the measurement properties of the instruments as well as estimate the minimum clinically important differences and responder thresholds for the PRO and ObsRO instruments.

There are a number of notable limitations to this study. First, participants were chosen from one metropolitan area only, which could limit generalizability of the study findings. In addition, the diagnosis of conjunctivitis was not confirmed by cultures. In clinical practice, a lack of effective antiviral treatments and the self-resolving nature of bacterial infection limit the value of routine cultures or other diagnostic testing to establish the underlying cause of infection and potential treatment response. Thus, clinicians often rely on signs and symptoms for diagnosis and/or may prescribe antibacterial agents on an empiric basis for suspected or equivocal cases, which can lead to inappropriate use of antibiotics. Another limitation of this study is that conclusions drawn are based on a small sample size. In particular, there were only 4 children in the dyads (aged 9–10 years). Despite this, concept saturation was achieved well within the sample size; similar terms were used by children, adolescents and adults to describe their symptoms; and interpretation of the questions was consistent across rounds. In addition, the participants aged 9–10 years had no difficulties in understanding or answering the questions during the cognitive interviews. While there were no participants aged 8 years in our study sample, the literature suggests that children aged ≥8 years are able to self-report on symptoms such as pain [14, 15]. We therefore recommend that children aged 8–17 years should be instructed to answer the PRO questions themselves, without any assistance or influence from their caregiver. For children aged < 8 years, the ObsRO should be completed by the caregivers to capture observable signs indicative of how the child feels.

Participants were asked to recall their experiences retrospectively in this study. Future research to evaluate these instruments prospectively is needed and would help to track changes in symptoms over the course of the illness. Research to validate incremental changes of symptoms using the instruments and their correlation with clinician-graded objective signs would also be useful. Results from the use of the instruments in clinical trials should provide information to address these questions and validate the instruments in the context of clinical trials of infectious conjunctivitis. Further development of the instruments should also include psychometric evaluation in a larger sample of patients with infectious conjunctivitis.

## Conclusions

This study supports the inclusion of items on pain, itching and foreign body sensation for a PRO and a general item on eye pain or discomfort for an ObsRO to evaluate ocular symptoms associated with viral or bacterial conjunctivitis in adult and pediatric patients.

## Data Availability

The datasets used and/or analyzed during the current study are available from the corresponding author on reasonable request.

## Abbreviations

ClinRO: Clinician-reported outcome
COA: Clinical outcome assessments
DEX: Dexamethasone
FDA: US Food and Drug Administration
ObsRO: Observer-reported outcome
PerfOs: Performance outcomes
PRO: Patient-reported outcome
